# Current status of prevention and treatment of respiratory diseases in primary care in China: a cross-sectional study

**DOI:** 10.1186/s12890-022-01956-6

**Published:** 2022-04-24

**Authors:** Xueqin Chen, Wei Sun, Shan Li, Xinmin Wang, Mao Huang, Ningfei Ji

**Affiliations:** 1 Department of Respiratory and Critical Care Medicine, The First Affiliated Hospital of Nanjing Medical University, Nanjing, 210029 China; 2 Department of Respiratory and Critical Care Medicine, Xishan People’s Hospital of Wuxi City, Wuxi, 214000 Jiangsu Province China; 3 Department of Respiratory and Critical Care Medicine, The Affiliated Jiangning Hospital of Nanjing Medical University, Nanjing, 211100 China; 4 Department of Respiratory and Critical Care Medicine, Shuyang Hospital of Traditional Chinese Medicine, Suqian, 223699 Jiangsu Province China

**Keywords:** Respiratory department, Chronic respiratory diseases, Primary health care, PHC

## Abstract

**Background:**

China launched its new round of health care reform to develop primary care in 2009, establishing 954,390 primary care institutions that employed over 10 million staff by 2019. However, some studies have shown that the prevention and management of respiratory diseases is inadequate in these institutions.

**Methods:**

We conducted a cross-sectional survey of grassroots institutions throughout China between September and December 2020 based on the standardized Prevention and Treatment System and Capacity Building Project of Respiratory Diseases in primary care settings. The operation of the respiratory department in primary health care institutions was evaluated in terms of facilities, drugs, personnel and management of chronic diseases by means of questionnaires. Descriptive analyses were performed to calculate percentages and frequencies of key parameters.

**Results:**

A total of 144 primary health care institutions were surveyed, including 51 in the east, 82 in the west, 9 in the central and 2 in the northeast. Approximately 60% of institutions had spirometers and pulse oximeters. The majority had short-acting bronchodilators, theophylline, systemic corticosteroids, antibiotics, and traditional Chinese medicine. More than half had at least one respiratory physician and operator for spirometry. Half of the institutions carried out screening of chronic obstructive pulmonary disease within the jurisdiction. The institutions in the east were superior to those in the west regarding the equipment, common drugs, medical staff, and management of respiratory diseases.

**Conclusions:**

The study reveals that the overall operation of the respiratory department in primary care settings needs to be further strengthened. It is crucial to provide adequate essential equipment, medical professionals, and medicines for proper diagnosis and treatment of chronic respiratory diseases, as well as improving the management of diseases.

**Supplementary Information:**

The online version contains supplementary material available at 10.1186/s12890-022-01956-6.

## Introduction

Primary health care (PHC) is a health development strategy established by the World Health Organization to achieve the goal of “health for all”, also known as the Declaration of Alma-Ata [1]. As the first level of contact with the national health system, primary health care aims to provide integrated, accessible basic health care to individuals and families in the community [1, 2]. In China, the development of primary health care has been difficult. The main direction of health care reform in the late 1980s was to give hospitals autonomy rather than receiving funding from public finance (fiscal decentralization); consequently, the gap between urban and rural areas widened, and health care costs soared [3, 4]. Confronted with these challenges, the government launched a new health care reform in 2009 [3] and established five action plans that were proven to make primary care more accessible and affordable. Furthermore, government subsidies to PHC institutions have increased more than tenfold (from ¥19 billion in 2008 to ¥197 billion in 2018) [5–7]. As of 2019, China has set up 954,390 primary medical institutions that employ more than 10 million health professionals [8].

Grassroots institutions are responsible for the first diagnosis, screening, treatment and referral of common noncommunicable diseases (NCDs) for the largest population of patients and high-risk groups. Chronic respiratory diseases, one of the four major NCDs, are responsible for 10.7% of NCD deaths [9]. However, some studies showed that grassroots institutions were not performing their proper role as gatekeepers, especially for chronic respiratory diseases. Wang and colleagues [10] found that most people with chronic obstructive pulmonary disease (COPD) were unaware of their condition, and few had received a previous pulmonary function test. Asthma, identified by the Global Burden of Disease Study as being the most globally prevalent chronic respiratory disease, was largely undiagnosed and undertreated in China, which might be attributed to the underdevelopment of primary care services [11, 12]. In addition, it was found that the management of chronic respiratory diseases was not as good as that of diabetes and hypertension in a sample survey of PHC institutions in Weifang, Shandong Province [13]. Under these circumstances, the standardized Prevention and Treatment System and Capacity Building Project of Respiratory Diseases in PHC institutions, led by the Respiratory Physician Branch of the Chinese Medical Doctor Association and Respiratory Society of Chinese Medical Association, was launched in September 2019 to promote the prevention and management of respiratory diseases in PHC institutions in China.

The situation of prevention and treatment of respiratory diseases is becoming increasingly severe in the context of the current global coronavirus disease (COVID-19) pandemic. Nevertheless, little data are available for the operation of the respiratory department in primary care settings. Hence, we conducted this study to explore the current operation of the respiratory department in PHC institutions in China, including material and human resources, as well as the management of respiratory diseases.

## Methods

### Study design

This was a cross-sectional study of primary health care (PHC) institutions in China. Between September and December 2020, we collected questionnaire-based data on the respiratory department in primary care settings throughout China based on the standardized Prevention and Treatment System and Capacity Building Project of Respiratory Diseases in PHC institutions. According to the standards of the National Bureau of Statistics in China, grassroots institutions were classified into four regions: east, west, central and northeast.

The questionnaire covered infrastructure, medicine, staff and management of chronic respiratory diseases in grassroots institutions. The full score of the questionnaire was 100 points, and the above four aspects accounted for 26%, 18%, 20%, 36% of the score, respectively. A final score ≥ 80 was regarded as “excellent unit”, scores ≥ 60 and < 80 (given the difference in economic development between eastern and western regions, western region < 75) were regarded as “standard unit”, and scores < 60 were regarded as “cultivation unit”.

### Statistical analysis

Continuous variables are presented as the mean ± standard deviation (${\overline{\text{x}}}$ ± s) or median (interquartile range, IQR), and categorical variables are expressed as absolute values along with percentages. The equipment and medication for chronic respiratory diseases, medical staff specialized in respiratory medicine, and management of respiratory diseases in PHC institutions were shown as categorical variables. For the comparison of facilities, drugs, staff and management of chronic diseases among different regions, we used the Kruskal–Wallis test, χ^2^ test, or Fisher’s exact test where appropriate. Logistic regression models were used to estimate the odds ratios (ORs) and 95% confidence intervals (CIs) for the association between the units and categorical outcomes. All significance tests were 2-tailed, and those with a *p* value < 0.05 were considered statistically significant. All statistical analyses were performed with SPSS, version 26.0.

## Results

**Fig. 1:**
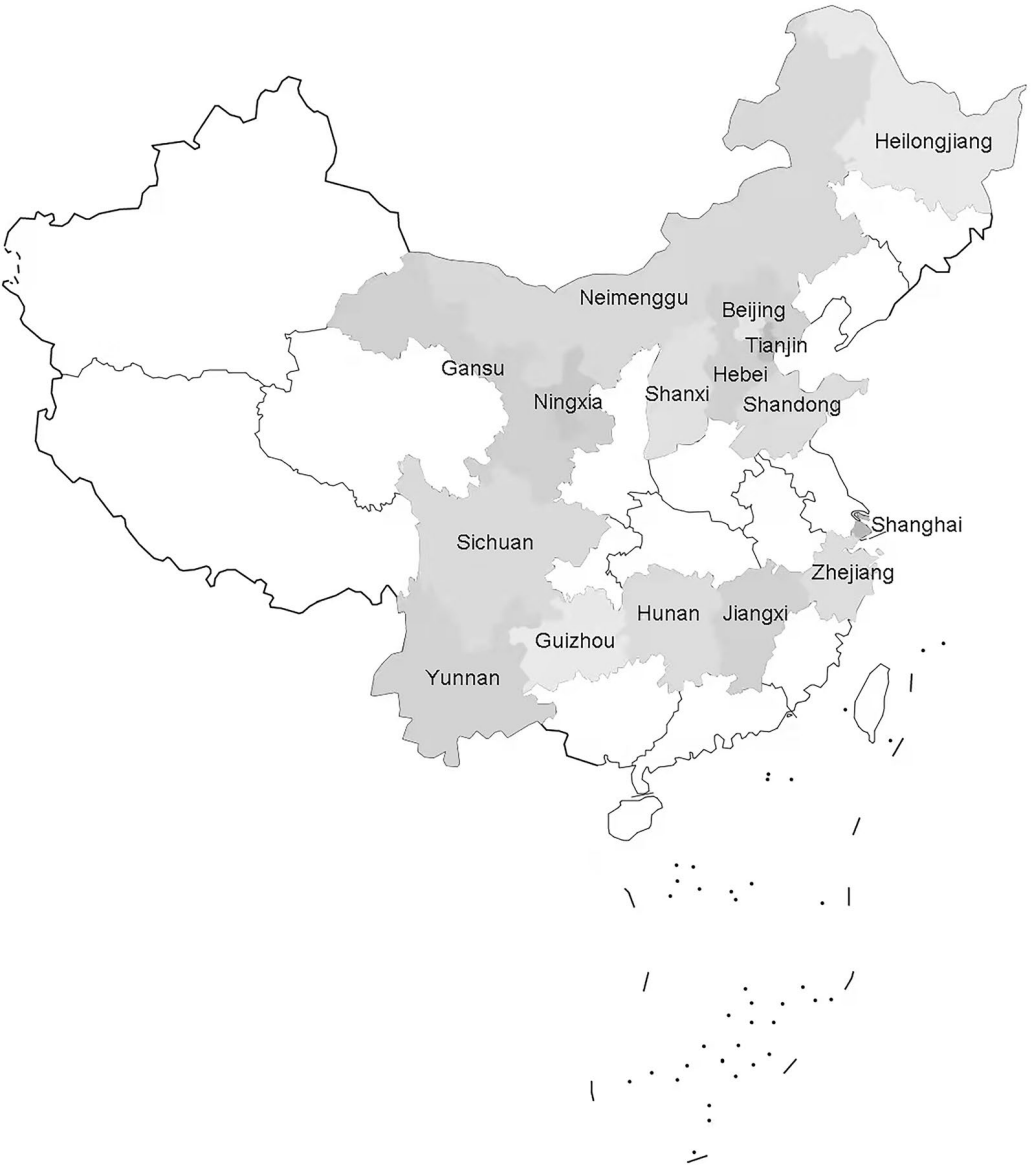
Distribution of primary health care institutions surveyed across China

A total of 144 PHC institutions (including community health centers or stations and township health centers) were involved, including 51 in the east, 82 in the west, 9 in the central and 2 in the northeast. The distribution of the surveyed PHC institutions is shown in Fig. 1.

As illustrated in Table 1, the self-construction period (the time from the establishment of PHC institutions until now) was 10 (5.25–25) months, and the standardized self-score was 60.75 (23–69) points. There were no excellent units, and 37 units reached the standard, accounting for 25.7%. Of 144 PHC institutions, 80% were equipped with infrastructure for the diagnosis and treatment of respiratory diseases, 91% were equipped with commonly used drugs, 69.4% had medical personnel specialized in respiratory medicine, and 73.6% had carried out the diagnosis and management of chronic respiratory diseases.

**Table 1:** General characteristics of the primary health care institutions (N = 144)

Characteristic	Value
Types of institutions	
Community health centers, No. (%)	29 (20.1%)
Community health stations, No. (%)	7 (4.9%)
Township health centers, No. (%)	108 (75%)
Self-establishment period, median (IQR)	10 (5.25–25)
Standardized self-score, median (IQR)	60.75 (23–69)
Institutions equipped with diagnostic facilities for respiratory diseases, No. (%)	115 (79.9%)
Institutions equipped with basic respiratory medication, No. (%)	131 (91%)
Institutions equipped with medical staff specialized in respiratory medicine, No. (%)	100 (69.4%)
Institutions which carried out diagnosis and management of respiratory diseases, No. (%)	106 (73.6%)
Classification of institutions, No. (%)	
Excellent units	0
Standard units	37 (25.7%)
Cultivate units	107 (74.3%)

*IQR* interquartile range

Table 2 shows the material resources of the PHC institutions. Briefly, 60% of institutions had spirometers and pulse oximeters. Three-quarters of them were equipped with nebulization devices, blood cytometers, X-ray machines and oxygen therapy equipment. More than half had separate areas for pulmonary function examination and atomization treatment. Most institutions had short-acting bronchodilators, theophylline, systemic corticosteroids, antibiotics, and traditional Chinese medicine. However, the supply of leukotriene receptor antagonist (LTRA) and combination low dose inhaled corticosteroids—long-acting beta2 agonist (ICS-LABA) drugs was relatively lacking (close to or less than 55%).

**Table 2:** The equipment and medication for chronic respiratory diseases in PHC institutions

Characteristic	Value
Equipment, No. (%)	
Spirometer	91 (63.2%)
Number of nebulization equipment	
> 3	58 (40.3%)
2–3	33 (22.9%)
1	20 (13.9%)
0	33 (22.9%)
Nebulizer mask for children	97 (67.4%)
Pulse oximeter	91 (63.2%)
Hematology analyzer	113 (78.5%)
X-ray apparatus	105 (72.9%)
Oxygen therapy device	108 (75%)
Size of the area for pulmonary function test	
≥ 8 m^2^	72 (50%)
< 8 m^2^ and > 0	8 (5.6%)
0	64 (44.4%)
Size of the area for nebulization	
≥ 8 m^2^	77 (53.5%)
< 8 m^2^ and > 0	8 (5.6%)
0	59 (40.9%)
Medicine, No. (%)	
SAMA/SABA	116 (80.6%)
LAMA/LABA	88 (61.1%)
Combination low dose ICS -LABA	79 (54.9%)
Theophylline	127 (88.2%)
LTRA	72 (50%)
Systemic corticosteroid	126 (87.5%)
Antibiotics	131 (91%)
Traditional Chinese medicine	126 (87.5%)

*SAMA* short-acting muscarinic antagonist, *SABA* short-acting beta2 agonist, *LAMA* long-acting muscarinic antagonist, *LABA* long-acting beta2 agonist, *ICS* inhaled corticosteroids, *LTRA* leukotriene receptor antagonist

Table 3 shows the human resources of the PHC institutions. The same proportion of institutions (56.3%) had at least one respiratory physician and operator for spirometry. Two-thirds were staffed with nurses or technicians for nebulization. With respect to scientific effort, sizable shares of institutions did not lead or participate in research related to the prevention and treatment of respiratory diseases at the grassroots level, let alone publish related papers.

**Table 3:** Medical staff specialized in respiratory medicine and their scientific effort in PHC institutions

Characteristic	Value
Respiratory medical staff, No. (%)	
Respiratory physicians	
≥ 2	61 (42.4%)
1	20 (13.9%)
0	63 (43.7%)
Operators for spirometry	
> 2	47 (32.6%)
1–2	34 (23.6%)
0	63 (43.7%)
Nurses/technicians for nebulization	
> 2	61 (42.4%)
1–2	33 (22.9%)
0	50 (34.7%)
Scientific effort, No. (%)	
Research projects related to PTRD in charge	
> 2	1 (0.7%)
1–2	3 (2.1%)
0	140 (97.2%)
Research projects related to PTRD involved in	
> 2	3 (2.1%)
1–2	12 (8.3%)
0	129 (89.6%)
Published papers on PTRD	
> 2	5 (3.5%)
1–2	7 (4.9%)
0	132 (91.7%)

*PTRD* prevention and treatment of respiratory diseases at the grassroots level

Table 4 illustrates the situation of diagnosis and management of respiratory diseases in PHC institutions. More than 200 cases of pulmonary function tests and nebulizer inhalation were performed annually in 16% and 27% of institutions, respectively. Half of the institutions conducted COPD screening in the community. Less than 40% had at least one case of asthma or COPD per year according to outpatient medical records. Contracted family physician services were provided in over half of the institutions. Approximately 60% carried out dual referral of respiratory diseases with secondary and tertiary hospitals.

**Table 4:** Diagnosis and management of respiratory diseases in PHC institutions

Characteristic, No. (%)	Value
Cases of pulmonary function tests per year	
0	71 (49.3%)
1–50	36 (25%)
51–200	14 (9.7%)
> 200	23 (16%)
Cases of aerosol inhalation therapy per year	
0	62 (43.1%)
1–100	35 (24.3%)
101–200	8 (5.6%)
> 200	39 (27.1%)
COPD screening for residents in the areas under their jurisdiction	74 (51.4%)
Outpatient cases of asthma per year	
0	95 (65.9%)
1–200	45 (31.3%)
> 200	4 (2.8%)
Outpatient cases of COPD per year	
0	86 (59.7%)
1–200	47 (32.6%)
> 200	11 (7.6%)
Family practice contract services (asthma)	
0	70 (48.6%)
1–100	64 (44.4%)
> 100	10 (6.9%)
Family practice contract services (COPD)	
0	59 (41%)
1–100	65 (45.1%)
> 100	20 (13.9%)
Public health education related to respiratory diseases	101 (70.1%)
Joining regional medical alliances of secondary and tertiary hospitals	101 (70.1%)
Bidirectional referral with secondary and tertiary hospitals	92 (63.9%)

Table 5 details differences in PHC institution characteristics according to the 2 regions. PHC institutions in the eastern region were better equipped and used than those in the western region overall (*p* < 0.05).

**Table 5:** Differences in PHC institution characteristics according to region

Characteristic (No. (%))	East (n = 51)	West (n = 82)	*p* East versus West
Equipment			
Spirometer	48 (94.1%)	33 (40.2%)	*p* < 0.001
Nebulization equipment	49 (96.1%)	51 (62.2%)	*p* < 0.001
Independent area for spirometry	43 (84.3%)	31 (37.8%)	*p* < 0.001
Pulse oximeter	45 (88.2%)	38 (46.3%)	*p* < 0.001
Medicine			
SAMA/SABA	47 (92.2%)	58 (70.7%)	0.003
Combination low dose ICS -LABA	39 (76.5%)	33 (40.2%)	*p* < 0.001
LTRA	41 (80.4%)	26 (31.7%)	*p* < 0.001
Staff			
Respiratory physicians	42 (82.3%)	32 (39.1%)	*p* < 0.001
Operators for spirometry	40 (78.4%)	35 (42.7%)	*p* < 0.001
Diagnosis and management			
Spirometry	40 (78.4%)	29 (35.4%)	*p* < 0.001
Nebulizer therapy	40 (78.4%)	37 (45.1%)	*p* < 0.001
COPD screening	36 (70.6%)	34 (41.5%)	0.001
Outpatien asthma cases	30 (58.8%)	17 (20.7%)	*p* < 0.001
Outpatient COPD cases	33 (64.7%)	20 (24.4%)	*p* < 0.001
Public health education	44 (86.3%)	53 (64.6%)	0.006
Joining regional medical alliances	43 (84.3%)	52 (63.4%)	0.009
Dual referral with superior hospitals	42 (82.4%)	45 (54.9%)	0.001

**Fig. 2:**
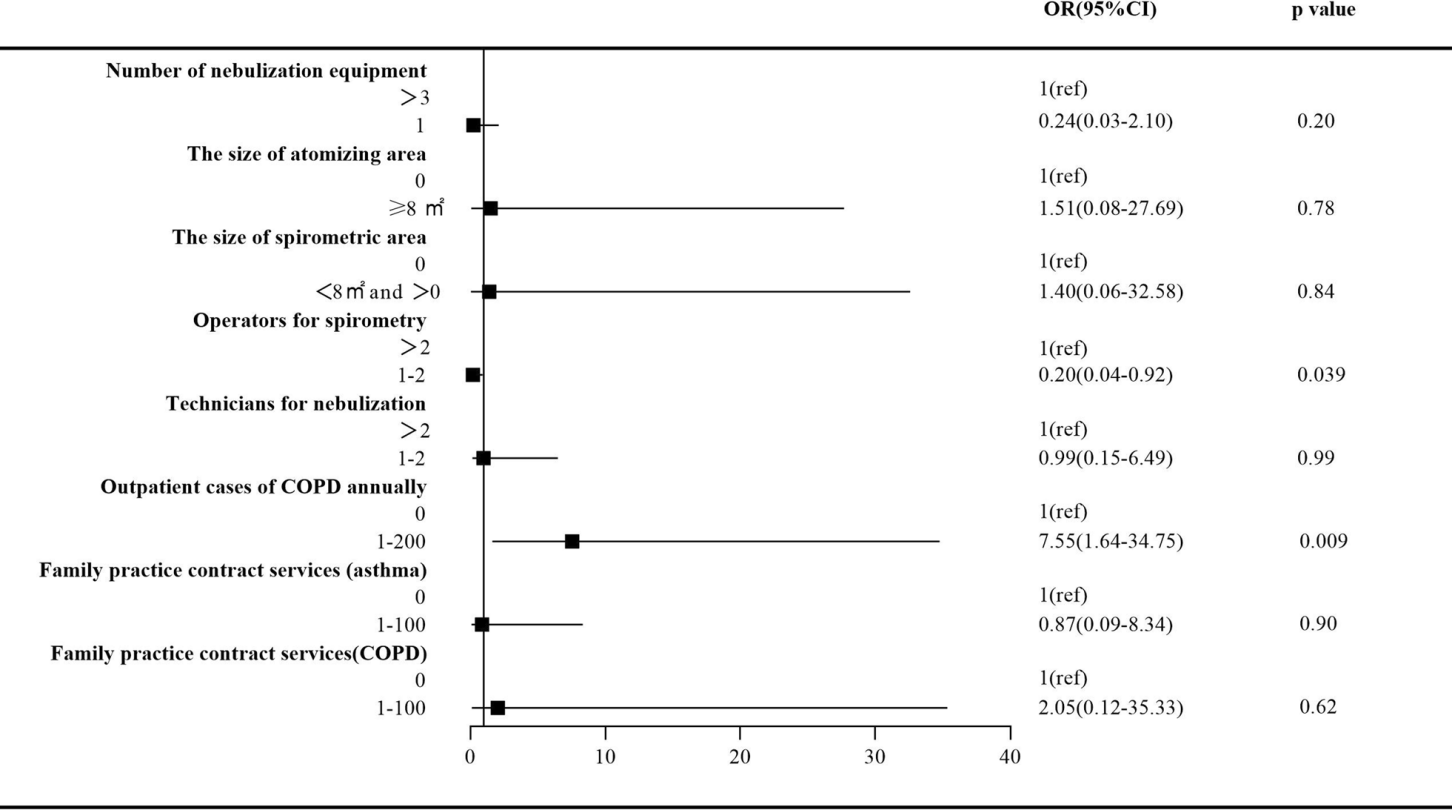
PHC institution characteristics associated with standard units

In unadjusted analysis, standard units were statistically significantly associated with nebulization equipment, the size of atomizing area, the size of the spirometric area, operators for spirometry, technicians for nebulization, outpatient cases of COPD annually, and family practice contract services (asthma or COPD) (*p* < 0.05; Additional file 1: Table S1). After multivariable adjustment, institutions having 1 or 2 operators for spirometry showed an OR of 0.20 (95% CI 0.04–0.92) for standard units compared with institutions having more than 2 operators (Fig. 2). In addition, institutions having 1 to 200 cases of COPD per year showed an OR of 7.55 (95% CI 1.64–34.75) compared with those void of outpatient cases of COPD (Fig. 2). The remaining six variables were not significant.

## Discussion

The government has introduced a series of policies to reinforce the prevention and treatment of chronic respiratory diseases in recent years. The Healthy China 2030 Plan, the national roadmap, included chronic respiratory diseases represented by COPD in the chronic disease prevention plan in 2019 [14]. To our knowledge, this is the first study to systematically evaluate the operation of respiratory specialties in primary care in China.

The material resources in PHC institutions in our study were satisfactory in general. However, as essential equipment for the diagnosis of most chronic respiratory diseases, spirometers are not available in a fair number of institutions, thus making the operation of pulmonary function tests impossible and making the diagnosis of some respiratory diseases, especially COPD, difficult. Although Abbasi et al. [15] suggested that validated respiratory questionnaires such as the ATS questionnaire could serve as a useful adjuvant to spirometry for screening respiratory diseases in community settings of resource-poor countries, it has yet to be widely implemented in clinical practice. Fortunately, the government has subsidized ¥1 billion for the first time to equip 50% of PHC institutions with spirometers in September 2020. Regarding medications, there was a relative lack of long-acting bronchodilators and combination low-dose ICS-LABA compared with short-acting bronchodilators and systemic corticosteroids. We hypothesize that primary care physicians would prefer to use the latter because of their rapid onset and significant effect. Not surprisingly, many PHC institutions had antibiotics and traditional Chinese medicine for respiratory diseases. Nevertheless, it is necessary to be aware of the risk of dual infection or super-resistant bacteria caused by antibiotic abuse. Wang et al. [16] reported that 52.9% of the outpatient visit prescription records in 48 PHC facilities in China contained antibiotics, but only 39.4% were prescribed properly.

As opposed to material resources, these institutions were not well staffed with respiratory physicians. Nonetheless, some studies demonstrate that nonphysician health workers can serve many aspects of primary care functions, including diagnosis and management, with equal or greater reliability and at lower cost [17, 18]. A positive correlation was also found between the high activity of community health workers and PHC practices targeting hypertension, diabetes, children’s health and women's health [19]. Of note, we found that having less than two operators for spirometry was a risk factor for standard units; that is, being equipped with more operators for spirometry may make it easier for the institutions to reach the standard. However, many primary care physicians do not have access to spirometry, as in our study, or may have difficulty with its performance and interpretation [20]. In addition to inadequate staffing, the scientific research force is rather weak, possibly due to limited access, lack of provider training, and time constraints in a busy practice setting. A previous survey showed that continuing education for PHC doctors was insufficient, with 36% of them not receiving continuing training courses in the past year [21].

The management of respiratory diseases in grassroots medical institutions appears to need much improvement. Cases of COPD and asthma, the two most common chronic respiratory diseases, were present in an outpatient setting in less than half of the institutions. Apart from the actual lack of diagnostic equipment or respiratory physicians, this may be partly due to the general public’s perception of PHC institutions as “poorly equipped and of low quality” [22]. They may prefer superior hospitals and seek medical treatment from specialists. Wong et al. [23] found that community health centers providing more services and longer working hours were not more acceptable and used to a greater extent by the masses. Interestingly, institutions having 1 to 200 outpatient cases of COPD annually were more likely to be a standard unit than those without outpatient cases, slightly reflecting the importance of chronic disease outpatient service. Family practice, often considered the core of primary care, is associated with better quality of care and lower medical costs [24]. Kuang et al. [24] further reported that the improved patient-perceived quality of care was related to the contract with family practice physicians, which may be a gateway to improve the public’s perceptions of primary care services. Obviously, the institutions providing family practice contract services for asthma or COPD in our study were far from sufficient. Additionally, there was an increase in the proportion of institutions conducting screening of COPD compared to a previous study [13], which benefited from policy support to a great extent.

The huge gap between the eastern and western regions reflects the uneven distribution of medical resources, consistent with economic trends. On this basis, inefficient use of medical resources may aggravate the disparity.

In view of the above problems, further improvements can be made as follows. First, the government should continue its ongoing efforts to establish a chronic respiratory disease management system. An increase in medical subsidies to western regions may be needed to reduce the imbalance of medical resources. Simultaneously, it requires concerted efforts by governments, health authorities and grassroots medical institutions to effectively integrate medical resources within the region. The training of respiratory professionals and technicians should be consolidated at the level of policy and institutions. Moreover, PHC institutions should set up chronic disease clinics for asthma and COPD, provide family practice contract service and carry out screening for chronic diseases, as well as health education in the community. Last, a discrete choice experiment revealed that preferences for primary care were significantly influenced by the provision of home visits, the distance to clinics, the clinics’ opening hours, and the diagnostic facilities in clinics [25]. As a consequence, PHC doctors could take the initiative to provide home visits, especially for the vulnerable group of older, multimorbid and immobile persons who have specific needs concerning care provision, or PHC doctors could conduct telephone interviews to facilitate monitoring of patients’ medication and symptoms.

This study has several limitations. First, only a relatively small number of institutions are included, which could result in a wide confidence interval. The scoring criteria for the questionnaire are applicable to China, whereas the specific evaluation and quantitative standards for other countries and regions depend on the circumstances. Finally, patient perceptions are widely studied in developed countries and considered to be one of the pillars of health care quality, on par with clinical effectiveness and patient safety [26, 27]. However, our study did not include this factor.

## Conclusion

Our study preliminarily reveals the current situation of prevention and treatment of respiratory diseases in primary care: insufficiency of some facilities and medicines, shortage of respiratory professionals, and irregular management of chronic respiratory diseases. In future studies, a larger sample size and better design are needed to further explore the operation of the respiratory department in primary care in China.

## Supplementary Information

**Additional file 1.** Association of risk factors with standard units.

## Data Availability

The datasets generated and/or analysed during the current study are not publicly available because the project mentioned in the paper is still in progress, but they are available from the corresponding author on reasonable request.

## Abbreviations

CI: Confidence interval
COPD: Chronic obstructive pulmonary disease
COVID-19: Coronavirus disease 2019
ICS: Inhaled corticosteroids
IQR: Interquartile range
LABA: Long-acting beta2 agonist
LAMA: Long-acting muscarinic antagonist
LTRA: Leukotriene receptor antagonist
NCDs: Noncommunicable diseases
OR: Odds ratio
PHC: Primary health care
PTRD: Prevention and treatment of respiratory diseases at the grassroots level
SABA: Short-acting beta2 agonist
SAMA: Short-acting muscarinic antagonist
