# Supplementary material for: Current status of prevention and treatment of respiratory diseases in primary care in China: a cross-sectional study
Source: BMC Pulm Med. 2022 Apr 24;22:156. doi: 10.1186/s12890-022-01956-6 (PMC9035273; doi:10.1186/s12890-022-01956-6)
Supplement: Supplementary file 1 — Additional file 1. Association of risk factors with standard units. [file 12890_2022_1956_MOESM1_ESM.docx]

Table S1 Association of risk factors with standard units

|  | Total  (n=144) | Standard units  (n=37) | Cultivate units  (n=107) | p | Univariate regression model  OR (95% CI) |
| --- | --- | --- | --- | --- | --- |
| Number of nebulization equipment |  |  |  |  |  |
| ＞3  1 | 58/144(40.3%)  20/144(13.9%) | 25/37(67.6%)  3/37(8.1%) | 33/107(30.8%)  17/107(11.8%) | .032 | 1  0.23(0.06-0.88) |
| The size of atomizing area |  |  |  |  |  |
| 0  ≥8㎡ | 59/144(40.9%)  77/144(53.5%) | 2/37(5.4%)  32/37(86.5%) | 57/107(53.3%)  45/107(42.1%) | .00 | 1  20.27(4.61-89.12) |
| The size of spirometric area |  |  |  |  |  |
| 0  ＜8㎡ and ＞0 | 64/144(44.4%)  8/144(5.5%) | 2/37(5.4%)  2/37(5.4%) | 62/107(57.9%)  6/107(5.6%) | .032 | 1  10.33(1.23-87.09) |
| Operators for spirometry |  |  |  |  |  |
| ＞2  1-2 | 47/144(32.6%)  34/144(23.6%) | 29/37(78.4%)  8/37(21.6%) | 18/107(16.8%)  26/107(24.3%) | .001 | 1  0.19(0.07-0.51) |
| Technicians for nebulization |  |  |  |  |  |
| ＞2  1-2 | 61/144(42.4%)  33/144(22.9%) | 32/37(86.5%)  5/37(13.5%) | 29/107(27.1%)  28/107(26.2%) | .001 | 1  0.16(0.05-0.47) |
| Outpatient cases of COPD annually |  |  |  |  |  |
| 0  1-200 | 86/144(59.7%)  47/144(32.6%) | 5/37(13.5%)  26/37(70.3%) | 81/107(75.7%)  21/107(19.6%) | .00 | 1  20.06(6.87-58.51) |
| Family practice contract services (asthma) |  |  |  |  |  |
| 0  1-100 | 70/144(48.6%)  64/144(44.4%) | 6/37(16.2%)  25/37(67.6%) | 64/107(59.8%)  39/107(36.4%) | .00 | 1  6.84(2.58-18.14) |
| Family practice contract services (COPD) |  |  |  |  |  |
| 0  1-100 | 59/144(41.0%)  65/144(45.1%) | 2/37(5.4%)  27/37(18.8%) | 57/107(53.3%)  38/107(35.5%) | .00 | 1  20.25(4.55-90.20) |

OR=odds ratio. CI=confidence interval.
